# A Metabolomic Signature of Acute Caloric Restriction

**DOI:** 10.1210/jc.2017-01020

**Published:** 2017-09-28

**Authors:** Tinh-Hai Collet, Takuhiro Sonoyama, Elana Henning, Julia M. Keogh, Brian Ingram, Sarah Kelway, Lining Guo, I. Sadaf Farooqi

**Affiliations:** 1 University of Cambridge Metabolic Research Laboratories and National Institute for Health Research Cambridge Biomedical Research Centre, Wellcome Trust-Medical Research Council Institute of Metabolic Science, Addenbrooke’s Hospital, Cambridge, CB2 0QQ, United Kingdom; 2 Service of Endocrinology, Diabetes and Metabolism, Lausanne University Hospital, 1011 Lausanne, Switzerland; 3 Metabolon, Inc., Durham, North Carolina 27713

## Abstract

**Context:**

The experimental paradigm of acute caloric restriction (CR) followed by refeeding (RF) can be used to study the homeostatic mechanisms that regulate energy homeostasis, which are relevant to understanding the adaptive response to weight loss.

**Objective:**

Metabolomics, the measurement of hundreds of small molecule metabolites, their precursors, derivatives, and degradation products, has emerged as a useful tool for the study of physiology and disease and was used here to study the metabolic response to acute CR.

**Participants, Design, and Setting:**

We used four ultra high-performance liquid chromatography-tandem mass spectrometry methods to characterize changes in carbohydrates, lipids, amino acids, and steroids in eight normal weight men at baseline, after 48 hours of CR (10% of energy requirements) and after 48 hours of *ad libitum* RF in a tightly controlled environment.

**Results:**

We identified a distinct metabolomic signature associated with acute CR characterized by the expected switch from carbohydrate to fat utilization with increased lipolysis and *β*-fatty acid oxidation. We found an increase in *ω*-fatty acid oxidation and levels of endocannabinoids, which are known to promote food intake. These changes were reversed with RF. Several plasmalogen phosphatidylethanolamines (endogenous antioxidants) significantly decreased with CR (all *P* ≤ 0.0007). Additionally, acute CR was associated with an increase in the branched chain amino acids (all *P* ≤ 1.4 × 10^−7^) and dehydroepiandrosterone sulfate (*P* = 0.0006).

**Conclusions:**

We identified a distinct metabolomic signature associated with acute CR. Further studies are needed to characterize the mechanisms that mediate these changes and their potential contribution to the adaptive response to dietary restriction.

## Teaser

We conducted a metabolomic analysis of 770 small molecules in eight healthy lean men and identified a distinct metabolomic signature associated with acute caloric restriction.

The adipocyte-derived hormone leptin circulates at concentrations proportional to fat mass in weight-stable humans and is a pivotal regulator of energy homeostasis (1–3). Most individuals maintain a relatively stable body weight over long periods of time despite day-to-day fluctuations in the number of calories consumed and the amount of physical activity undertaken. Loss of fat mass leads to a fall in circulating leptin concentrations triggering changes in energy intake, energy expenditure, and neuroendocrine function that restore energy homeostasis, resulting in rebound weight gain—the phenomenon popularly known as yo-yo dieting. Rosenbaum *et al.* (4, 5) have shown that leptin administration in short-term human studies can reverse many of the consequences of weight loss (a state of partial leptin deficiency). However, recombinant leptin/leptin mimetics are not currently available for this purpose. The identification of additional markers of the homeostatic response to weight loss could inform therapeutic strategies to prevent weight regain and aid weight maintenance.

In seminal studies in mice, Ahima *et al.* (6) showed that, as well as being a marker of fat mass, leptin acts as a signal of nutrient availability; acute caloric restriction (CR)/starvation (without loss of fat mass) leads to a rapid fall in circulating leptin concentration, hyperphagia, reduced energy expenditure, and hypogonadism (6), features that mimic genetic leptin deficiency in rodents and humans (7, 8) and can be normalized by leptin administration (6, 9). Acute CR in humans can therefore be used as a model for investigating leptin-mediated homeostatic responses without the potential confounding effects of weight loss.

The metabolomic profile at a given time point represents the cumulative effects of the diet, genome, transcriptome, proteome, and gut microbiome–host interaction on small molecule metabolites whose concentrations change rapidly (10). By measuring the metabolome in the same individuals before and after a precise perturbation, our aim was to identify the metabolites that change significantly with acute CR and refeeding (RF). We assessed the short-term adaptation to CR to avoid confounding with metabolic changes related to weight loss.

We performed a carefully controlled experimental study to directly examine the effects of acute CR in eight normal weight young men housed in a clinical research facility where diet, fluid intake, timing of meals, and sleep were precisely controlled and monitored (Supplemental Table 1). Fasting blood samples were obtained at three time points: baseline (after 24 hours of feeding to normal energy requirements); after 48 hours of CR to 10% of their normal energy requirements [mean ± standard error of the mean (SEM), 226 ± 5 kcal/d]; after 48 hours of *ad libitum* RF to allow for energy homeostasis to be reset. Critically, the proportion of macronutrients received during CR was the same as on the baseline day (50% carbohydrate, 30% fat, 20% protein). In a previous study, we have shown that this experimental manipulation is sufficient to significantly alter physiological parameters including autonomic function, neuroendocrine hormone secretion, and the sleep/wake cycle (11). Here, in addition to measuring circulating hormones and peptides predicted to be altered by nutritional status (*e.g.,* leptin, insulin, glucagon, and thyroid hormones), we used a high throughput metabolomics assay employing four independent ultra high-performance liquid chromatography-tandem mass spectrometry (UPLC-MS/MS) methods to measure 770 small molecule metabolites involved in carbohydrate, fat, protein, and steroid metabolism.

## Material and Methods

### Experimental design of study

The study was approved by the Cambridge local research ethics committee and was conducted in accordance with the principles of the Declaration of Helsinki. Written informed consent was received from each participant prior to inclusion in the study. We recruited eight normal weight healthy men using the following inclusion criteria: normal glucose tolerance measured by a 75-g oral glucose tolerance test, no evidence of renal, liver, or thyroid disease, average alcohol intake <2 units/d, not participating in an organized exercise program, not treated with anorectic agents or medications known to affect carbohydrate and/or lipid metabolism, or blood pressure. Shift workers were excluded from the study; all participants had a normal sleep/wake pattern as determined by polysomnography at screening. Weight and height were measured barefoot in light clothing and body mass index calculated (weight in kg/height in meters squared). Baseline characteristics are provided in Supplemental Table 1.

Participants were resident on the National Institute for Health Research-Wellcome Trust Clinical Research Facility, Addenbrooke’s Hospital, Cambridge, United Kingdom, for the duration of the study under direct observation throughout. At baseline, volunteers consumed a balanced diet (50% carbohydrate, 30% fat, and 20% protein) matching their daily energy requirement calculated by the basal metabolic rate multiplied by a physical activity level of 1.25 using the Schofield Equation (12). To manipulate energy balance, baseline measurements were followed by CR to 10% of normal energy requirement (mean ± SEM, 226 ± 5 kcal/d) for 2 days. After CR, participants were offered three substantial *ad libitum* buffet meals per day (20 MJ = 4777 kcal) and additional snacks (16 MJ = 3821 kcal) between meals for 2 days. They were invited to eat freely until comfortably full; food consumption was covertly measured. Fluid intake was fixed for all participants; no coffee/tea/alcohol was permitted. All meals were given at the same time each day. Timing of “lights out” and waking were controlled. We collected fasting serum and plasma samples at 0800 am at baseline, after CR, and RF.

### Biochemical assays

Plasma glucose, insulin, glucagon, leptin, serum lipids, thyrotropin, free thyroxine (T4), and free tri-iodothyronine (T3) were measured. Testosterone and dehydroepiandrosterone sulfate (DHEAS) were measured with a DiaSorin Liaison XL analyzer (Saluggia, Italy), and 3-hydroxybutyrate using the Stanbio colorimetric kit (Boerne, TX). Blood samples were collected in the fasting state at 08:00 am.

### Metabolomic platform

The nontargeted metabolomic analysis was performed at Metabolon, Inc. (Durham, NC). All serum samples were stored at −80°C until processed. The detailed descriptions of the platform, including sample processing, instrument configuration, data acquisition, and metabolite identification and quantitation, were published previously (10) with the exception that four independent UPLC-MS/MS methods were used. The samples were extracted with methanol, and the supernatants were divided into five equal fractions for analysis by UPLC-MS/MS: (1) reverse phase (RP) UPLC-MS/MS method with positive ion mode electrospray ionization (ESI), optimized for more hydrophilic compounds. In this method, the extract was gradient eluted from a C18 column (Waters UPLC BEH C18-2.1 × 100 mm, 1.7 µm) using water and methanol, containing 0.05% perfluoropentanoic acid and 0.1% formic acid; (2) RP/UPLC-MS/MS method with positive ion mode ESI, optimized for more hydrophobic compounds. In this method, the extract was gradient eluted from the same aforementioned C18 column using methanol, acetonitrile, water, 0.05% perfluoropentanoic acid, and 0.01% formic acid and was operated at an overall higher organic content; (3) RP/UPLC-MS/MS method with negative ion mode ESI. In this method, the extract was gradient eluted from the column using methanol and water; however, with 6.5 mM ammonium bicarbonate at pH 8; (4) hydrophilic interaction liquid chromatography/UPLC-MS/MS with negative ion mode ESI. In this method, the extract was gradient eluted using water and acetonitrile with 10 mM ammonium formate, pH 10.8; and (5) reserved for backup. For UPLC-MS/MS, all methods used a Waters ACQUITY UPLC (Waters Corp., Milford, MA) and a Thermo Scientific Q-Exactive high resolution/accurate mass spectrometer (Thermo Fisher Scientific, Waltham, MA) interfaced with a heated ESI-II source and Orbitrap mass analyzer operated at 35,000 mass resolution. All the methods alternated between full scan mass spectrometry (MS) and data-dependent MS^n^ scans using dynamic exclusion. The scan range varied slighted between methods but generally covered 70 to 1000 m/z.

The structure of metabolites was identified by automated comparison of the ion features in the experimental samples to a reference library of chemical standard entries that included retention time, molecular weight (m/z), preferred adducts, and in-source fragments, as well as associated MS spectra and curated by visual inspection for quality control using software developed at Metabolon (13–15).

### Statistical analyses

#### Statistical analyses of biochemistry results

Log-transformed values were analyzed using analysis of variance with repeated measures to test for within-subject changes across the three study conditions (baseline, CR, RF). The within-subjects *P* value was adjusted using the Greenhouse-Geisser correction factor for lack of sphericity. Pairwise comparisons of the three study conditions were performed by two-sided Student *t* test when appropriate. A *P* value of 0.05 was considered significant after Bonferroni correction for multiple comparisons.

#### Statistical analyses of metabolite semiquantitative levels

Following scaling of the ion count (so that the median equals 1), imputation of any missing values with the minimum observed value for each metabolite and log transformation, analysis of variance contrasts and Welch matched-pair *t* tests were used to identify biochemicals that differed significantly between experimental groups. Multiple comparisons were accounted for with the false discovery rate method (16). An estimate of the false discovery rate (*q* value) was calculated to take into account the multiple comparisons that normally occur in metabolomic-based studies. A low *q* value (*q* < 0.10) is an indication of high confidence in a result. Although a higher *q* value indicates diminished confidence, it does not necessarily rule out the significance of a result. Principal component analysis (PCA) and hierarchical clustering of scaled, imputed, and log-transformed metabolite levels are detailed in the Supplemental Methods. Statistical analyses were performed using JMP (SAS Institute, Inc., Cary, NC) and R software (R Foundation, Vienna, Austria).

## Results

### A metabolomic signature of CR

Acute CR was associated with a significant fall in circulating fasting leptin, insulin, and glucose concentrations and an increase in glucagon levels (Table 1). We noted a decrease in free T3 but no significant change in thyrotropin or free T4 in response to acute CR as seen in some previous studies (17, 18). Most parameters returned to baseline values with RF, whereas others (insulin, glucagon) were sensitive to the overconsumption of calories on *ad libitum* RF and exceeded baseline values (Table 1).

**Table 1.** **Changes in Fasting Biochemistry in CR and Upon RF**

**Mean (SEM)**	**Baseline**	**CR**	**RF**	***P* Values for Overall Comparison**			
				**Overall**	**BL-CR**	**CR-RF**	**BL-RF**
Caloric intake, kcal/d	2255 (53)	226 (5)	4552 (324)	<0.0001	<0.001	<0.001	<0.001
Glucose metabolism*^a^*							
Glucose, mmol/L	4.74 (0.12)	3.48 (0.08)	4.70 (0.11)	<0.0001	<0.001	<0.001	1.00
Insulin, pmol/L	44.6 (7.7)	14.6 (2.6)	87.6 (14.2)	<0.0001	<0.001	<0.001	<0.001
Glucagon, pg/mL	24.8 (2.7)	74.7 (11.6)	38.2 (4.2)	0.0001	<0.001	<0.001	0.01
3-OH butyrate, µmol/L	101.8 (7.8)	1763 (258)	93.7 (7.4)	<0.0001	<0.001	<0.001	1.00
Leptin, ng/mL	3.26 (0.81)	0.66 (0.19)	4.55 (1.34)	<0.0001	<0.001	<0.001	0.22
Lipid metabolism*^b^*							
Total cholesterol, mmol/L	4.11 (0.34)	4.30 (0.29)	3.73 (0.27)	0.0009	0.18	<0.001	0.006
Triglycerides, mmol/L	1.31 (0.11)	1.24 (0.10)	1.52 (0.14)	0.10			
Thyroid function*^c^*							
TSH, mU/L	1.34 (0.18)	1.14 (0.19)	2.00 (0.29)	0.006	0.20	0.001	0.04
Free T4, pmol/L	13.93 (0.35)	14.80 (0.51)	14.94 (0.47)	0.03	0.07	1.00	0.03
Free T3, pmol/L	5.02 (0.19)	3.92 (0.17)	4.65 (0.28)	0.001	<0.001	0.002	0.12
Steroids*^c^*							
Testosterone, nmol/L	18.51 (2.10)	17.05 (2.52)	15.27 (2.12)	0.10			
DHEAS, µg/dL	312.2 (43.8)	347.1 (39.9)	298.6 (49.7)	0.10			

Data are presented as mean (SEM). We tested the correlation of biochemistry assay concentrations with levels measured by metabolomics; the Pearson correlation ranged between *ρ* = 0.87 and *ρ* = 0.95 (for glucose, 3-OH butyrate and DHEAS) but was lower for cholesterol (*ρ* = 0.70; the metabolomics platform measures free cholesterol, whereas the standard clinical assay measures total cholesterol) and free T4 (*ρ* = 0.50).

Abbreviations: 3-OH butyrate, 3-hydroxybutyrate; BL, baseline; TSH, thyroid-stimulating hormone.

^a^ To convert glucose values to milligrams per deciliter, multiply by 18. To convert 3-OH butyrate values to milligrams per deciliter, divide by 96.09. Reference ranges for insulin: 0 to 60 pmol/L; 3-OH butyrate: 20 to 270 µmol/L.

^b^ To convert lipid values to milligrams per deciliter, multiply by 38.7 for total cholesterol and 88.5 for triglycerides.

^c^ Reference ranges for TSH: 0.35 to 5.5 mU/L; free T4: 10.0 to 19.8 pmol/L; free T3: 3.5 to 6.5 pmol/L; testosterone: 8.0 to 29.0 nmol/L; DHEAS: 161.2 to 561.6 µg/dL.

**Figure 1.**
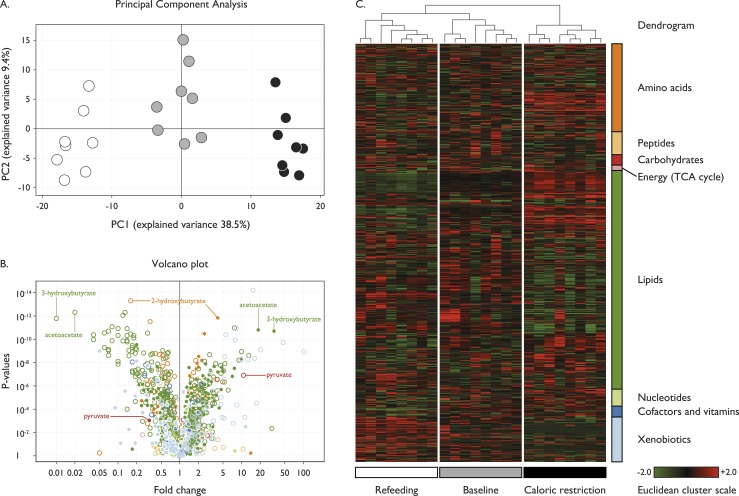
Metabolomic signature of CR. Seven hundred seventy metabolites were measured in eight subjects at three time points: baseline (gray), after CR (black), and upon RF (white). (A) The PCA; principal component 1 (PC1) captured 38.5% of the variance of the data set and discriminated well between the three study conditions, whereas component 2 (PC2) covered 9.4% of the variance. (B and C) Changes in metabolite categories: amino acids (dark orange), peptides (light orange), carbohydrates (red), energy and the tricarboxylic acid (TCA) cycle (pink), lipids (dark green), nucleotides (light green), cofactors and vitamins (dark blue), and xenobiotics (light blue). (B) A volcano plot of the statistical significance as indicated by *P* values (*y*-axis) associated with fold change in each metabolite (*x*-axis); baseline to CR (filled circles); CR to RF (open circles). A large percentage of metabolites significantly increased (38%; n = 295) or decreased (39%; n = 300) upon CR. (C) A heatmap derived from hierarchical clustering of the metabolomic data. Clustering was performed using complete linkage and Euclidean distance, where each sample is a vector with all of the metabolite values. The color scale correlates with relative metabolite abundance across the samples: the black indicates median value, red an elevation above the median, and green a decrease below the median.

We explored the metabolomic profile for each participant at baseline, CR, and in the refed state by employing multivariate statistical analyses to analyze state-dependent changes. PCA was conducted on log-transformed metabolite levels to identify whether aggregated values of specific components could account for a proportion of the variability between baseline, CR, and RF. We found that the PCA of the metabolome completely distinguished the three states revealing a characteristic metabolomic signature associated with CR (Fig. 1A). While within a state, there was variability between participants (presumably due to genetic/biological factors), the magnitude and direction of effect of CR and RF was comparable for all participants (Supplemental Fig. 1).

A substantial proportion of the entire metabolome exhibited a dynamic response to CR and RF (Fig. 1B). We used hierarchical clustering to identify large-scale differences in metabolite categories for each individual in each state. The resulting heatmap revealed that CR induced a number of changes in lipid and carbohydrate utilization; some of which were reversed upon RF (Fig. 1C). Further discussion is focused on metabolites that constitute this dynamic metabolomic signature. The complete data set is presented in Supplemental Table 2 and an associated interactive webpage is available (www.goos.org.uk).

### Carbohydrate utilization: glycolysis and the tricarboxylic acid cycle

**Figure 2.**
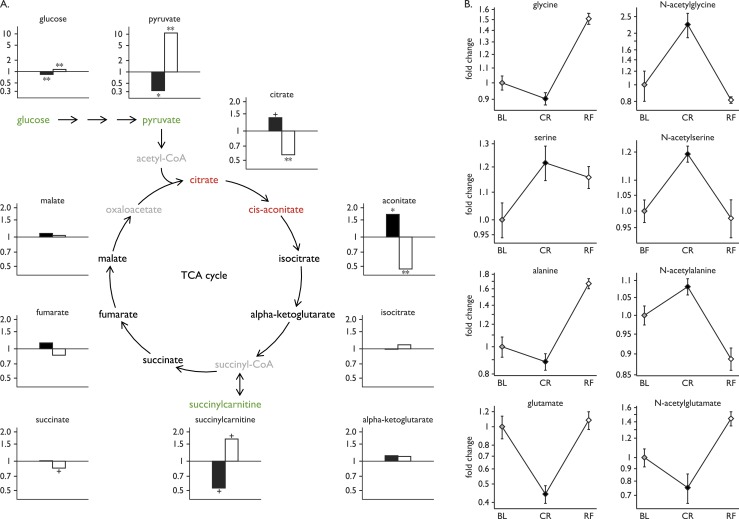
Glycolysis and gluconeogenesis. (A) Statistically significant increases (red) and decreases (green) in metabolites involved in glycolysis and the TCA cycle with CR (black bars) and upon RF (white bars). Fold changes in glucose, pyruvate, and TCA cycle components are shown (*y*-axis). Some metabolites were not measured in this assay (gray). Statistical significance is presented as follows: + for *P* values between 0.001 and 0.05; * for *P* values ≤ 0.001; ** for *P* values ≤ 0.001 and *q* values ≤ 0.001. (B) Fold changes in glucogenic amino acids and their *N*-acetyl-derivatives during the study. Plots show mean ± SEM of the eight subjects at baseline (BL; gray), CR (black), and RF (white).

Classically, glycolysis converts glucose to pyruvate, which, under aerobic conditions, is converted into acetyl-CoA, the entry point into the tricarboxylic acid (TCA) cycle (Fig. 2A). As expected, CR was associated with a decrease in glucose (fold change 0.84, *P* = 5.9 × 10^−5^) and pyruvate (fold change 0.32, *P* = 9.0 × 10^−4^), which were restored by RF (fold change 1.14 and 10.8, *P* = 8.0 × 10^−4^ and 1.2 × 10^−7^, respectively) (Fig. 2A). Citrate and aconitate were significantly elevated with CR, there was no change in isocitrate or *α*-ketoglutarate, but a significant decrease in succinylcarnitine, an intermediate arising reversibly from succinyl-CoA (Fig. 2A). *N*-acetyl derivatives of the glucogenic amino acids glycine, serine, and alanine increased with CR and decreased with RF (Fig. 2B). In contrast, glutamate and its product *N*-acetylglutamate decreased with CR (Fig. 2B). These changes reversed upon RF and collectively are consistent with an increase in metabolite flux through the TCA cycle in response to CR.

Although some *N*-acetylated amino acid derivatives followed the same pattern across baseline–CR–RF conditions as their corresponding amino acids (*e.g.,* glutamate, serine), others did not (*e.g.,* glycine, alanine; Fig. 2B). The reasons for the differential effects of CR on *N*-acetylation of gluconeogenic amino acids need further investigation.

### Lipolysis and fatty acid oxidation

**Figure 3.**
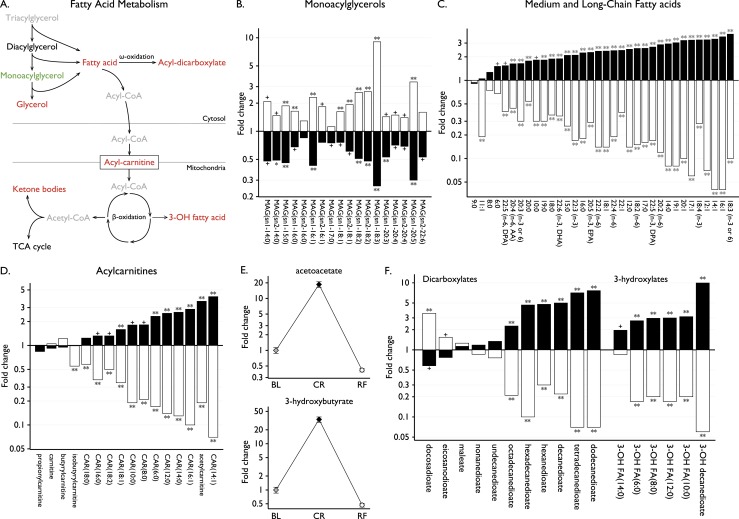
Fatty acid oxidation and lipolysis. (A) Metabolites involved in lipolysis and fatty acid oxidation that significantly increased (red) or decreased (green) with CR. Some metabolites were not measured in this assay (gray). (B–D) Fold changes in (B) monoacylglycerols (MAG), (C) medium and long-chain fatty acids, and (D) acylcarnitines (CAR) with CR (black) and RF (white). Lipid species are annotated using the following convention: lipid class [number of carbon atoms]:[number of double bonds], [position of double bond(s) if known]. In addition, for MAG, sn1 or sn2 indicate the esterification position in the glycerol backbone, which links the acyl group. Some fatty acids are also designated by abbreviations, such as arachidonic acid (AA), docosahexaenoate (DHA), docosapentaenoate (DPA), and eicosapentaenoate (EPA). (E) Fold changes in ketone bodies between baseline (BL), CR, and RF; mean ± SEM. (F) Fold changes with CR (black) and RF (white) in other lipid species involved in fatty acid oxidation. Statistical significance is presented as follows: + for *P* values between 0.001 and 0.05; * for *P* values ≤ 0.001; ** for *P* values ≤ 0.001 and *q* values ≤ 0.001.

CR was characterized by lipolysis of triglycerides generating increased levels of glycerol (2.43-fold, *P* = 0.0003), decreased monoacylglycerols, and an increase of all long and most medium chain fatty acids detected (Fig. 3A–3C). During CR, we found a significant increase in long-chain acylcarnitines, which shuttle fatty acids into mitochondria (Fig. 3D). Consequently, the ketone bodies acetoacetate and 3-hydroxybutyrate, final products of the fatty acid *β*-oxidation pathway, were markedly increased with CR (*P* = 1.6 × 10^−11^ and *P* = 2.0 × 10^−11^, respectively) and decreased with RF (*P* = 4.9 × 10^−13^ and 1.6 × 10^−12^, respectively) (Fig. 3E). Serum levels of ketone bodies were measured in routine laboratory tests for comparison; 3-hydroxybutyrate increased 17.3-fold (Table 1).

Additionally, 3-hydroxy fatty acids were increased in keeping with increased lipolysis and fatty acid *β*-oxidation (Fig. 3F). Furthermore, fatty acid dicarboxylates, in particular dodecanedioate and tetradecanedioate, which increased 7.7-fold on CR, are generated through the *ω*-oxidation pathway (Fig. 3F). Although this pathway has rarely been studied in humans, our study suggests that activation of *ω*-oxidation may occur when *β*-oxidation becomes overwhelmed as it may in CR. Taken together, CR resulted in increased reliance on fatty acid (rather than carbohydrate) metabolism to fuel energy production. RF led to a decreased reliance on fatty acid oxidation to meet energetic needs and stimulation of lipogenesis (Fig. 3B–3F).

### Phospholipids and endocannabinoids

**Figure 4.**
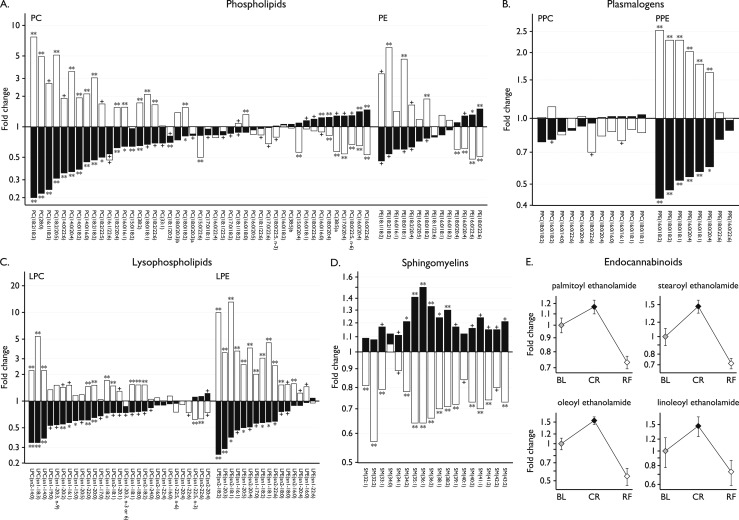
Phospholipids and other lipid mediators. Fold changes in phospholipids and other lipid mediators with CR (black) and RF (white): (A) glycerophospholipids, (B) plasmalogens, (C) lysophospholipids, and (D) sphingomyelins. The acyl group(s) are specified using the following convention: [number of carbon atoms]:[number of double bonds], [position of double bond(s) if known]; or the total numbers if unknown. In addition, for LPC and LPE, sn1 or sn2 indicate the esterification position in the glycerol backbone, which links the acyl group. PC(18:0/20:3)_a_ and PC(18:0/20:3)_b_ are structurally similar but differ by the position of the double bonds. The precise structure of PC (38:5)_a_ and PC(38:5)_b_ could not be specified. Statistical significance is presented as follows: + for *P* values between 0.001 and 0.05; * for *P* values ≤ 0.001; ** for *P* values ≤ 0.001 and *q* values ≤ 0.001. (E) Fold changes in endocannabinoids between baseline (BL), CR, and RF; mean ± SEM. LPC, lysophosphatidylcholine; LPE, lysophosphatidylethanolamine; PC, phosphatidylcholine; PE, phosphatidylethanolamine; PPC, plasmalogen phosphatidylcholine; PPE, plasmalogen phosphatidylethanolamine; SM, sphingomyelin.

Although triglycerides represent the major source of stored lipid, phospholipids are the major constituents of plasma membrane lipids and eicosanoids, such as prostaglandins, leukotrienes, and thromboxanes. We found that most glycerophospholipids and their product lysolipids were decreased in CR and increased upon RF (Fig. 4A–4C). Plasmalogens are a subclass of glycerophospholipids that serve as endogenous antioxidants, protecting membrane lipids and lipoprotein particles from excessive oxidation by scavenging reactive oxygen species via the vinyl ether moiety (19, 20). Interestingly, the plasmalogen phosphatidylethanolamines, but not the plasmalogen phosphatidylcholines, decreased on CR and increased with RF (Fig. 4B).

Sphingolipids play a role in the formation of membrane lipid rafts, are components of plasma lipoprotein associated particles, and act as ligands for specific cell-surface receptors. In our study, the most abundant sphingolipids, the sphingomyelins, increased with CR; this pattern was reversed in the refed state (Fig. 4D). Sphingosine, sphingosine-1-phosphate, and ceramides are interconvertible sphingolipid metabolites; many studies have shown that their relative levels regulate cell fate (21, 22). Levels of sphingosine (generally associated with growth arrest and cell death) decreased in CR (0.52-fold, *P* = 0.03), whereas there was no change in sphingosine-1-phosphate, which is involved in cell growth and survival.

Another group of lipid signaling molecules, the endocannabinoids, modulate energy intake and expenditure by acting on central neural pathways expressing the cannabinoid 1 receptor, a target of the weight-loss agent rimonabant (23). Although there was no change in 2-arachidonoyl glycerol, the most abundant endogenous ligand of cannabinoid receptors, which has a very short half-life (Supplemental Table 2), all other detected endocannabinoids increased in CR then decreased upon RF (Fig. 4E). These findings are consistent with experimental evidence in animals where a key role for endogenous endocannabinoids is to stimulate food intake (24).

### Amino acids and their derivatives

**Figure 5.**
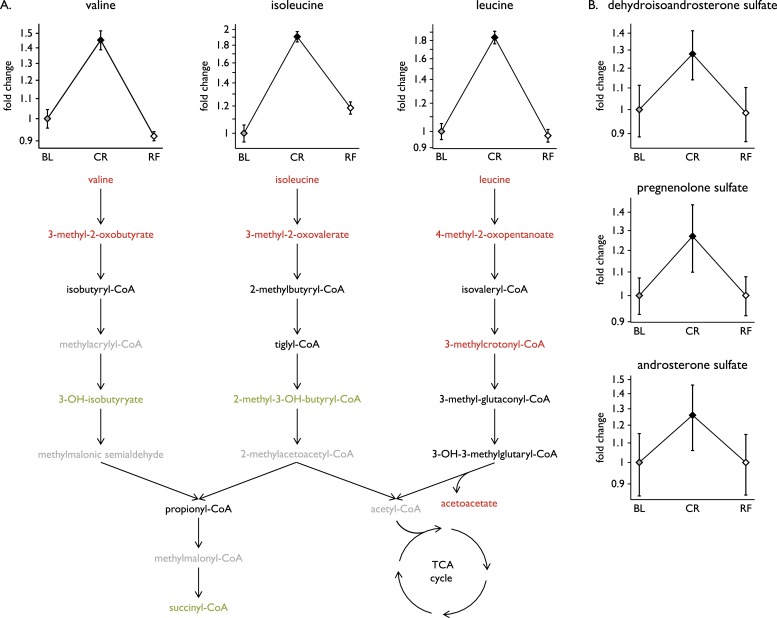
Amino acids and steroid hormones. (A) Fold changes in the BCAAs valine, isoleucine, and leucine between baseline (BL), CR, and RF; mean ± SEM. Steps in BCAA catabolism are shown; metabolites that significantly increased (red) or decreased (green) with CR are indicated. Some metabolites were not measured in this assay (gray). (B) Fold changes in a subset of steroid hormones between conditions; mean ± SEM.

Several amino acids and their metabolites (in particular tryptophan and its derivatives) decreased in CR, then increased with RF (Fig. 1C; Supplemental Table 2); these changes may in part reflect reduced dietary intake. In contrast, the three-branched chain amino acids (BCAAs), leucine, isoleucine, and valine significantly increased upon CR (all *P* ≤ 1.4 × 10^−7^; Fig. 5A). BCAAs are degraded by the branched-chain *α*-keto acid dehydrogenase complex into acyl-CoA derivatives, which are converted into acetyl-CoA or succinyl-CoA that enter the TCA cycle. We observed increased levels of many BCAA catabolites consistent with accelerated BCAA catabolism under CR which reversed upon RF (Supplemental Fig. 2).

### Steroid metabolism

In contrast to cortisol, which changes minimally with age, circulating concentrations of the adrenal steroid hormone DHEAS are high during early adulthood then decline markedly with age (25). Some, but not all, studies in primates and humans (26) have suggested that chronic CR may affect DHEAS levels. In this study, 48 hours of CR was associated with an increase in DHEAS and the steroid hormones pregnenolone and androsterone; values returned to baseline levels on RF (Fig. 5B; Supplemental Fig. 3). Cortisol levels did not change significantly during the study (Supplemental Table 2).

## Discussion

Using an experimental design in which we stabilized dietary intake across participants for 24 hours before an observed period of acute CR, followed by *ad libitum* RF to restore energy homeostasis, we identified a characteristic metabolomic signature associated with acute CR. Some of our findings align with previous studies that have measured targeted sets of metabolites in response to an overnight/prolonged fast (27, 28). Although changes in glycolysis and markers of fatty acid *β*-oxidation were predicted, our study shows that *ω*-oxidation (generally regarded as a minor fatty acid oxidation pathway in humans) may be activated in response to acute CR in healthy individuals. *ω*-oxidation is used as part of the compensation for defective *β* or *α*-fatty acid oxidation in rare genetic disorders, such as Refsum disease and X-linked adrenal leukodystrophy (29).

We saw consistent class-wide changes in several lipid species (phospholipids, sphingolipids, and endocannabinoids), which may reflect the mobilization of fatty acids for energetic purposes. In keeping with this hypothesis, CR/RF induced changes in fatty acids correlated with changes in their respective fatty acyl carnitines (Supplemental Fig. 4). Additionally, we observed a negative correlation between the change in fatty acid levels and the change in lysophosphatidylcholines with CR (Supplemental Fig. 5), an effect that may be mediated by neuropathy target esterase-related esterase, an enzyme which has lysophospholipase activity, is expressed in adipose tissue and skeletal muscle, and is upregulated upon fasting (30).

By focusing on the 27 long chain fatty acids and polyunsaturated fatty acids examined in this study (Fig. 3C), we observed that palmitoleate (16:1), a major constituent of the glycerides in human adipose tissue that has been associated with increased insulin sensitivity (31), exhibited a highly significant change with CR (3.61-fold, *P* = 1.9 × 10^−5^). In contrast, arachidonate, a major *ω*-6 fatty acid and precursor of lipid-derived mediators of inflammation (leukotrienes and thromboxanes) changed least in response to CR (Fig. 3C). Thus, although the whole scale changes in fatty acids we observed during CR represent the activation of fatty acid oxidation to supply energetic needs, specific changes in fatty acids may predispose to increased insulin sensitivity and reduced cellular inflammation, findings which have potential relevance to protection from aging (32, 33) and require further exploration.

In this study, we identified a series of metabolite changes associated with acute CR without weight loss. The changes in carbohydrate, lipid, and amino acid metabolism that we observed are characteristic of the counter-regulatory response that acts to defend against starvation and restore energy homeostasis. Although this degree of CR is seldom experienced by most people, short-term CR for weight loss engages the same physiological and biochemical mechanisms. The activation of these mechanisms, whose function is to restore energy balance, underpins weight regain, the phenomenon popularly known as yo-yo dieting. The application of metabolomics analyses to carefully controlled studies of weight loss and the comparison with the data set reported here, may reveal biomarkers that predict the degree of weight loss/regain.

Experimental studies in animal models have consistently shown that CR, typically 40% to 60% of energy requirements, can extend lifespan by up to 50% compared with *ad libitum* fed animals (34–36). Our study permits comparison with a large body of research into the effects of CR in lower organisms, although there are challenges in comparing data across species and from serum rather than cells/tissues (37, 38). One particularly interesting convergence is the finding that some phosphatidylethanolamines, lipids that are known to affect cell survival and the lifespan of yeast and flies (39), decreased with CR and increased with RF.

In conclusion, we have shown that analysis of the metabolome in a carefully conducted clinical study can provide insights into the mechanisms underpinning physiological processes, such as the response to acute CR in humans. With replication, these studies have the potential to provide valuable insights into the mechanisms underlying the effects of CR, which may inform new therapeutic opportunities for weight maintenance.
