# Supplementary material for: A Metabolomic Signature of Acute Caloric Restriction
Source: J Clin Endocrinol Metab. 2017 Sep 28;102(12):4486–95. doi: 10.1210/jc.2017-01020 (PMC5718701; doi:10.1210/jc.2017-01020)
Supplement: Supplementary file 2 [file jc.2017-01020.st1.docx]

# Online Supplementary Material

### Supplementary Methods

#### Principal components analysis and hierarchical clustering. Principal components analysis (PCA) is an unsupervised analysis that reduces the dimension of the data, where each principal component is a linear combination of every metabolite. Hierarchical clustering is another unsupervised method for clustering the data and can show large-scale differences. We used complete linkage clustering using the Euclidean distance, where each sample is a vector with all of the metabolite values. We plotted a heat map of the clustering results with the metabolites colored according to their relative abundance across the samples: black for median value, red an elevation above the median, and green a decrease below the median.

### Supplementary Table 1. Participant characteristics at baseline

|  | **Mean (SEM)** |
| --- | --- |
| **Age, years** | 24.5 (1.9) |
| **Sex** | All male |
| **Height, m** | 1.79 (0.02) |
| **Weight, kg** | 75.9 (2.8) |
| **Body mass index** ^a^**, kg/m^2^** | 23.6 (0.6) |
| **Blood pressure and heart rate** |  |
| Systolic blood pressure, mmHg | 118.8 (4.4) |
| Diastolic blood pressure, mmHg | 64.8 (4.6) |
| Heart rate, beats/min | 54.5 (2.1) |
| **Body composition** ^b^ |  |
| % body fat | 20.6 (2.4) |
| Fat mass, kg | 15.3 (2.3) |
| Lean mass, kg | 57.3 (1.5) |
| **Lipid profile** ^c^ |  |
| Total cholesterol, mmol/l | 4.20 (0.31) |
| Triglycerides, mmol/l | 1.03 (0.15) |
| High-density lipoprotein cholesterol, mmol/l | 1.14 (0.06) |
| Low-density lipoprotein cholesterol, mmol/l | 2.64 (0.28) |
| **75-gram oral glucose tolerance test** ^d^ |  |
| Fasting plasma glucose, mmol/l | 4.56 (0.11) |
| Plasma glucose at 120 min, mmol/l | 5.36 (0.59) |

Footnotes: Data are presented as mean values and standard error of the mean (SEM). ^a^ Body mass index is weight in kilograms divided by the height in meters squared. ^b^ Body composition was measured by dual energy X-ray absorptiometry. ^c^ To convert lipid values to milligrams per deciliter, multiply by 38.7 for cholesterol levels and 88.5 for triglycerides. ^d^ To convert glucose values to milligrams per deciliter, multiply by 18.

### Supplementary Table 2. Complete metabolomics dataset across conditions

See Excel spreadsheet uploaded separately.

Footnote:

Compounds containing a “*” are compounds where an authentic standard was not readily available. These “*” identifications are based on predictable retention properties and similar mass spectral data characteristics as compared to authenticated compounds in the Metabolon library of similar compound class and structure.

### Supplementary Figure 1. Principal component analysis with individual markers and lines

Legend: Seven hundred and seventy metabolites were measured in eight subjects at three timepoints. Changes in each subject are shown: baseline (gray), after caloric restriction (black) and upon refeeding (white). Principal component 1 (PC1) captured 38.5% of the variance of the dataset and discriminated well between the three study conditions, while component 2 (PC2) covered 9.4% of the variance.

### Supplementary Figure 2. BCAA breakdown products

Legend: Most breakdown products of the branched chain amino acids (BCAA) increased in caloric restriction, black bars) and decreased upon refeeding (white bars). Statistical significance is presented as follows: + for p-values between 0.001 and 0.05; * for p-values ≤ 0.001; ** for p-values ≤ 0.001 with q-values ≤ 0.001.

### Supplementary Figure 3. Sterols and steroids

Legend: Most measured sterols and steroids increased in caloric restriction (black bars) and decreased upon refeeding (white bars). Statistical significance is presented as follows: + for p-values between 0.001 and 0.05; * for p-values ≤ 0.001; ** for p-values ≤ 0.001 with q-values ≤ 0.001.

### Supplementary Figure 4. Correlation of non-esterified fatty acids and acylcarnitines

Legend: Panel A shows the correlation between the fold change in long chain non-esterified fatty acids (NEFA, y-axis) and the fold change in their respective acylcarnitines (x-axis) with caloric restriction (linear regression of logged values: R^2^ = 0.89, p = 0.001). Panel B shows the correlation between the fold change of NEFAs and the fold change in their respective acylcarnitines upon refeeding (linear regression of logged values: R^2^ = 0.95, p = 0.001). Source data is included in Supplementary Table 3.

### Supplementary Figure 5. Correlation of non-esterified fatty acids and lysophosphatidylcholines

Legend: Panel A shows the correlation between the fold change in long chain non-esterified fatty acids (NEFA, y-axis) and the fold change in their respective lysophosphatidylcholines (LPC, x-axis) with caloric restriction (linear regression of logged values: R^2^ = -0.52, p = 0.02). Panel B shows the correlation between the fold change of NEFAs and the fold change in their respective LPCs upon refeeding (linear regression of logged values: R^2^ = -0.46, p = 0.04). Source data for NEFAs and LPC sn1 forms is included in Supplementary Table 3.
